# Driver’s Cognitive Workload and Driving Performance under Traffic Sign Information Exposure in Complex Environments: A Case Study of the Highways in China

**DOI:** 10.3390/ijerph14020203

**Published:** 2017-02-17

**Authors:** Nengchao Lyu, Lian Xie, Chaozhong Wu, Qiang Fu, Chao Deng

**Affiliations:** 1 Intelligent Transportation Systems Research Center, Wuhan University of Technology, Wuhan 430063, China; lnc@whut.edu.cn (N.L.); xielian@whut.edu.cn (L.X.); woec@outlook.com (C.D.); 2 Engineering Research Center for Transportation Safety, Ministry of Education, Wuhan 430063, China; 3 School of Architecture and Transportation Engineering, Guilin University of Electronic Technology, Guilin 541004, China; 4 Traffic Management Research Institute of the Ministry of Public Security, Wuxi 214151, China; fqiangs@163.com

**Keywords:** complex environments, cognitive workload, traffic sign Information, driving simulator, driving characteristic

## Abstract

Complex traffic situations and high driving workload are the leading contributing factors to traffic crashes. There is a strong correlation between driving performance and driving workload, such as visual workload from traffic signs on highway off-ramps. This study aimed to evaluate traffic safety by analyzing drivers’ behavior and performance under the cognitive workload in complex environment areas. First, the driving workload of drivers was tested based on traffic signs with different quantities of information. Forty-four drivers were recruited to conduct a traffic sign cognition experiment under static controlled environment conditions. Different complex traffic signs were used for applying the cognitive workload. The static experiment results reveal that workload is highly related to the amount of information on traffic signs and reaction time increases with the information grade, while driving experience and gender effect are not significant. This shows that the cognitive workload of subsequent driving experiments can be controlled by the amount of information on traffic signs; Second, driving characteristics and driving performance were analyzed under different secondary task driving workload levels using a driving simulator. Drivers were required to drive at the required speed on a designed highway off-ramp scene. The cognitive workload was controlled by reading traffic signs with different information, which were divided into four levels. Drivers had to make choices by pushing buttons after reading traffic signs. Meanwhile, the driving performance information was recorded. Questionnaires on objective workload were collected right after each driving task. The results show that speed maintenance and lane deviations are significantly different under different levels of cognitive workload, and the effects of driving experience and gender groups are significant. The research results can be used to analyze traffic safety in highway environments, while considering more drivers’ cognitive and driving performance.

## 1. Introduction

Impaired driving behaviors caused by complex traffic situations are the leading contributing factors in many traffic crashes [1]. When the environment becomes more complex, drivers have to process this information, which lead to high driving workloads. Workload has been defined by Senders [2] as a measure of the effort expended by a human operator while performing a task, independently of the performance of the task itself. It shows the relationship between driving performance and driving workload. When workload levels are low, performance is also low, because of the information missed due to inattention. As the workload increases, the level of performance increases up to a maximum level. This maximum performance represents the optimal level of workload for a given task. Additional driving workload leads to an abrupt decrease in performance due to the large amount of information to be processed [3].

Previous studies concerning driving workload mainly focused on the measurement of driving behavior, especially the measurement of physiological and psychological parameters, such as EEG and HRV index; in addition, scholars have paid attention to quantitative measurement from the perspective of driving performance. This paper investigated drivers’ speed control and lane keeping characteristics under different cognition workload levels, and then quantified driving performance that could be used to assess driving workload. Moreover, in further study, the principle of how external environment variables affect driving workload and driving performance will be confirmed.

In terms of driving workload upload, previous studies mainly adopted N-back tasks or numerical calculation tasks, considering the advantages that the operation of experiments is relatively simple and the degree of complexity of the secondary task can controlled by subject evaluation, but the additional workload is not easy to quantify. The results of static experiments have revealed that workload was highly related to the amount of information on traffic signs and reaction time increases with the grade of information, while driving experience and gender effect are not significant. This shows that the cognitive workload of the subsequent driving experiment can be controlled by the amount information on traffic signs. In this study, an important consideration for the present work is that the information of traffic signs can be calculated, and participants were instructed to recognize traffic signs with different information. In addition, using traffic signs as the loading mode of driving workload is more in line with the characteristics of natural driving on highway off-ramp sections. The secondary task was used to load different levels of driving workload. Therefore, the driving performance under quantified workload can be obtained. The effect of additional workload of traffic signs on natural driving performance is the focus of the present study and the regularity of driving performance changes with different gender and driving experience groups of participants has implications for driving safety and traffic sign design.

### 1.1. Driving Workload under a Complex Environment

Driving workload changes in real time as the road environment changes. Inconsistent roadway design can produce unexpected changes in dynamic and speed conditions, which may impose high workloads that can surprise the driver and lead to operational errors. These inconsistencies, which should be controlled by the engineer, can result in critical driving maneuvers for motorists, and may increase the probability of an accident. American Association of State Highway and Transportation Officials (AASHTO) [1] noted that drivers’ reaction times increase as a function of decision complexity and the amount of information to be processed. Furthermore, the longer the reaction time, the greater the chance for errors.

Driving workload can be defined as the demand tasks exerted on a pool of undifferentiated mental resources. Research has shown that driving workload has a substantial influence on task performance [4]. When the driving workload is high, drivers may lose awareness of the importance of tasks. In this situation, primary driving task performance may be reduced. Research on the effects of workload on compliance with medical procedures has shown that high workload can reduce this compliance. When participants were working with chemical substances under low workload conditions, they were more likely to comply with warnings about wearing protective equipment while performing their task [5]. Other additional tasks such as telephone conversations while driving have a significant effect on driving workload, and consequently, on driving task performance [6]. Other studies have shown that distraction increases driving workload, as measured through a peripheral detection task [7].

Traffic signs are the main form of interaction of drivers with the environment, and the amount of information is the main factor that influences driving workload. Many previous studies have shown that traffic sign pictures, by way of collecting a driver’s reaction time, is an index to evaluate the traffic sign threshold for the number of road names [8,9,10,11]. For example, for road names from N to N + 1 road names, it is considered that the road name sign threshold is N, while reaction time significantly increased. Although this method reflects the visual recognition of the increase in driving time mutation load, it does not consider the situation when drivers are not able to withstand overload information, nor consider the impact of the actual driving speed and the driver’s reaction. As part of the road visual information, traffic signs also affect the visual characteristics of the driver; thus, affecting driving behavior and traffic safety. For example, under different visual environments under simulated driving conditions, a complex visual environment induces higher workload, lower driving speed and longer reaction time [12]. Benedetto et al. [13] studied changes in blink duration under different load conditions of vision. That study provides theoretical reference for the approach that prevents the driver from missing visual information. However, there is little quantitative research of visual information. Furthermore, there is no clear threshold of reasonable visual information. Visual workload can be controlled under certain levels, if the visual information and driving workload can be quantified by the information volume. The cognitive behavior of drivers under different environments and workloads also requires further study.

### 1.2. Driving Performance Analysis Based on Driving Experiments

Conducting a driving task experiment in the real world can explain behavior in different situations, such as geometric design, traffic operation, traffic signs, and so on. Previous research has shown that natural driving and field testing cars can be used to analyze the behaviors of drivers, such as driving when conversing on a cell phone [14,15], design inconsistencies on existing roads [16], and driving characteristics related to traffic accidents [17]. The real driving data provides more results compared with previous studies. However, it also has inherent shortcomings. Natural or field driving does not allow for control of the driving scene, such as when different traffic signs and road alignment are required. Moreover, drivers are faced with significant safety threats when driving in dangerous environments.

Due to the limited opportunity to study driving under different situations, one of the approaches used in studies was a driving simulator. Brookhuis and Waard [6] illustrated the potential of using modern high-standard driving simulator environments to monitor the driving workload of drivers during task performance. In driving simulators, measuring the driving workload of a driver can be conducted relatively easily by means of physiological measures and performance. Fitzpatrick [18] studied the notice and reaction time of drivers to a lead decelerating vehicle using a driving simulator. The objective of the study was to measure how long drivers took to notice and react to a lead decelerating vehicle. Hoogendoorn [19] performed two driving simulator experiments to investigate the influence of four factors of dynamic maximum speed limit signs on the perception, driving workload and compliance of drivers. Yan studied the effects of fog, driver experience and gender on driving behavior on S-curved road segments [20], as well as the effects of foggy conditions on the speed control behaviors of drivers at different risk levels [21]. All the above studies have shown the advantages and flexibility of using driving simulators in analyzing driving behaviors.

Research on the impact of driving workload is largely dependent on effective experimental methods and evaluation methods. With the constant improvement in driving behavior experiment platforms and analytical tools, the use of driving simulation scenarios and real field driving scenes to carry out an evaluation of the experiment is the current direction of driving workloads research. In this paper, simulated driving experiments were adopted to analyze driving performance.

### 1.3. Influence of Experience and Gender on Driving Performance

Studies have shown that experience and gender have an impact on driving performance. Moderate driving workloads can result in excellent driving performance, which is related to the driver’s learning process and experience [22]. In the present study, driver experience was measured by whether the driver was a professional driver or a non-professional driver. Compared with professional drivers, non-professional drivers consume more attention resources and cause higher workload [23], and their accident risk was 2–4 times higher [24,25]. Craen [26] illustrated that novice drivers generally misjudge the environment and the establishment of the required compensation strategy was also relatively slow, but they drove faster than skilled drivers although more driving skills were required in complex environments. Skilled drivers were adept in adjusting the vehicle lateral position to avoid the risk [27]. In addition, inexperienced drivers tend to have an elevated mental workload and inefficient visual search, hazard perception and vehicle control abilities [28].

Gender is one of the most often measured variables in driving behavior studies, and has been identified as a key demographic variable influencing driving performance. Studies in Europe have found that although females have a greater safety orientation than males, young female drivers have more problems in vehicle handling and mastering traffic situations [29]. It has long been believed that men are more likely to be involved in motor-vehicle crashes, however, female drivers are now over-represented in crashes compared to males, caused by errors in yielding, judging gaps and speed violations [30].

### 1.4. Objectives of This Study

Although additional cognitive load will be caused by traffic sign recognition [31], it remains unclear whether some quantitative characteristics for this kind of cognitive load exist with changes in traffic sign information, and whether there is a significant difference in the driver’s experience and gender to recognize traffic signs. In addition, the cognitive workload of traffic signs has some effects on the driver’s main task, which indicates the decline in performance [32]. Hence, there is concern on how driving performance changes in a complex cognitive environment. Furthermore, driver experience and gender should be given attention.

The detailed objectives of study 1 were: (1) to measure the reaction times of traffic signs with different information levels, and analyze the relation between traffic information volume and subjective workload rank; (2) to determine the effects of driver experience and gender on cognition time and subjective workload rank.

The detailed objectives of study 2 were: (1) to measure driving performance (mainly as reflected in the longitudinal speed control and lateral lane-keeping control) under different levels of additional cognitive workload caused by perceiving traffic signs as a secondary task; (2) to determine the effects of driver experience and gender on driving performance under different levels of driving workload.

## 2. Study 1: Relationships between Information Volume and Workload

The purpose of this experiment was to determine the effects caused by sign information changes through the analysis of behavioral data under different information workloads, thereby providing that more complex traffic sign information would produce a greater workload for drivers.

Traffic sign information is considered as an important factor that causes cognitive workloads; therefore, the relationship between traffic sign information volume and workload should be analyzed. The driving workload of drivers should be tested based on the information quantity of traffic signs.

Before the analysis and quantization of information workload, there is a need to quantify the factors that cause information workload. The key of information workload control is the amount of information in traffic signs. In order to obtain a different degree of workload, traffic signs with different amounts of information should be visually recognized. Information theory is used for the quantitative analysis of traffic sign information.

### 2.1. Quantification of Traffic Sign Information

The main function of traffic signs is to transfer traffic information to drivers. In cognitive psychology theory, a classic model of human behavior has been described as “stimulus-organism-response”. The traffic sign recognition process can be described in three stages: note, process, and decision. Traffic signs with different amounts of information can cause different information workloads on the driver. According to information theory, traffic sign information can be measured as follows [33]:

(1) $$H(X)=\sum_{i=1}^{n}H_{i}(X)=-\sum_{i=1}^{n}(\sum_{j=1}^{m}P(X_{j})\log_{2}P(X_{j}))$$

where *H*(*X*) is the amount of information of the traffic sign (bits); *H_i_*(*X*) is the quantity of information (bits) about elements which comprise the sign content, *n* is total number of element types; *X_j_* is the number *j* state of a certain object; *m* is total number of possible states of a certain object; *P*(*X_j_*) is the probability of the *j*-th state.

Assuming every state of an object will occur equally, the probability of every state would therefore be the same, that is, *P*(*X_j_*) = 1/m. Then, the method for calculating the amount of information can be simplified as follows:

(2) $$H_{i}(X)=\log_{2}m$$

Traffic signs contain different elements such as Chinese characters, English characters, numbers and different shaped characters. Furthermore, the amount of information for each element is different, and can be calculated by Equation (2). According to the “List of frequently used words in modern Chinese”, the number of Chinese characters used daily is about 3500, so the amount of information included for each Chinese characters is 11.77 bits ($H_{1}(X)=\log_{2}3500=11.77$). Similarly, the amount of information included for English characters, numbers, colors, arrows and symbols are 4.70, 3.32, 3.30, 4.64, 4.09 bits, respectively.

In the actual driving process, a driver’s perception of traffic elements is different. Wang [9] has conducted research on traffic element using the hierarchical analysis method, in which the weight of six kinds of elements was determined by five experienced drivers through the pairwise comparison of important traffic signs. The element weights are shown in Table 1.

In this study, the weight of six kinds of elements on road traffic signs was set based on the weight of Wang’s research results. The information amount in the calculation formula can be described using Equation (3):

(3) $$H(X)=\sum_{i=1}^{n}(a_{i}H_{i}n_{i})$$

where: *a_i_* is the weight of the element (refer to Table 1 for weight values); *H_i_* is the amount of information contained in a single element, calculated by Equation (2); *n_i_* is number of elements in traffic signs.

Liu [8] studied the visual recognition behavior of drivers towards different traffic signs based on this information calculation method. Self-reporting by drivers revealed that large amounts of information signs induce greater pressure on a driver’s vision and psychology, which produces a larger information cognitive workload. Some scholars have used this method to study drivers‘ visual recognition. For example, Wang [9] analyzed the relationship between understanding and information of drivers toward signs, based on the information calculation method. The results showed that cognitive difficulty increases as the amount of information increases. Furthermore, it also proves that the workload of signs with more information was larger than that of signs with a small amount of information.

In this study, the above information calculation method was employed to quantify traffic sign information, and analyze workloads with different amounts of information, on the basis of information quantification. Based on the principle that a driver’s reaction time for different traffic signs is different when reading traffic signs, reaction time and subjective workload rank were used to analyze and prove a driver’s driving workload variation with information variation.

### 2.2. Subjects

The experiment was a 4 (information level) × 2 (driving experience) × 2 (gender) repeated measures mixed design. Drivers were selected with the following driver experience requirements: at least 5000 km driving during the last year; for non-professional casual drivers, having held a driving license for at least 1 year and for professional taxi drivers, having a driving license for at least 3 years. Forty-four participants were recruited. The participants had no short-term health problems, according to their self-reporting. All participants had normal or corrected-to-normal vision. These participants were allocated into two groups, according to their profession: professional taxi drivers (*n* = 22; 17 males, 5 females), and non-professional casual drivers (*n* = 22; 14 males, 8 females). The professional drivers were full-time taxi drivers with an average driving experience of 13.5 years and an average annual driving distance of 81,200 km. The non-professional drivers used their vehicles for the purpose of daily travel only. Their average driving age was 3.32 years and average mileage was 10,600 km per year. The age of the participants ranged from 22 to 54 years (SD = 10.1) with an average of 35.6 years, and the average ages of the professional and non-professional groups were 42.2 and 28.8, respectively. All drivers participated both in Study 1 and Study 2.

### 2.3. Apparatus

**Figure 1:**
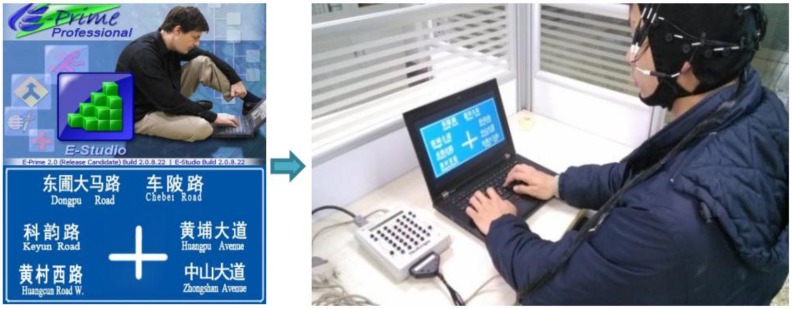
Apparatus and materials of Study 1.

The experiment was conducted using an E430 Lenovo computer in the Driving Behaviors Lab at Wuhan University of Technology. The computer had an Intel Corei5 processor and 2.0 GB memory. The display screen was a 23-inch LED screen with 1920 × 1080 resolution. The E-prime software (Experimenter’s Prime 2.0, Psychology Software Tools, Inc., Sharpsburg, MD, USA) in the computer was used to execute the experimental programs. The software can display materials selected by the pre-setup program, and can collect the keyboard or mouse operations of the participants. The experimental materials were a set of road traffic signs, which have different volumes of information. There were nearly 80 traffic signs designed by Auto CAD (Autodesk Computer Aided Design, 2007, Autodesk, Inc., San Rafael, CA, USA) and processed by Adobe Photoshop CS4 (Adobe Systems Incorporated, 2008, San Jose, CA, USA). The traffic signs were designed according to Road Traffic Signs and Markings (GB 5768.2-2009), with blue background and white text. Apparatus and materials of study 1 are shown in Figure 1.

### 2.4. Scenario Design and Data Collection

The 4 (information level) × 2 (driving experience) × 2 (gender) repeated measures mixed design presented four traffic sign information levels: level 1 (total information volume below 40 bits), level 2 (total information volume between 41 and 80 bits), level 3 (total information volume between 81 and 120 bits), and level 4 (total information volume above 121 bits). The experience and gender were the between-subjects factors and traffic sign information levels were the within subjects factors. The dependent variables were cognition time and subjective workload score. All participants (professional and non-professional drivers) participated in the four block experiment.

**Figure 2:**
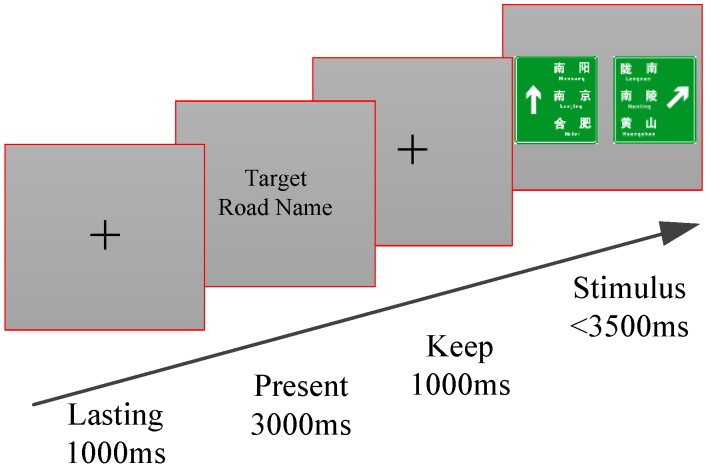
Procedure diagram of the test stimulus.

During the experimental process, the subjects were seated in front of the computer. Their eyes and the screen center maintained a distance of approximately 70 cm. When the experiment started, a target road name was displayed to the subjects first, with a rendering time of 3000 ms. This prompted the participants to find the target road name in the subsequent displayed signs. Then, a set of traffic signs that contained the target road name, as well as other distractions, would be displayed. The participants were required to recognize the target name and respond by pushing a keyboard button. After a participant’s response, the screen would turn white for 1000 ms before another traffic sign was displayed. Detailed demonstrations of the traffic sign sequence are shown in Figure 2. For each similar target road name, a total number of 7–8 traffic signs are required for subjects to recognize. In order to counterbalance the effect of the time order, traffic signs with different information levels were arranged in random sequence. After each block, the participant could take a one-minute rest. All participants had to finish four blocks, with four target road names and a total of 22 test traffic signs.

When participants watched the displayed traffic signs, they had to determine the location of the target road name as soon as possible, and click the related keyboard button. They had to press the “LEFT” button of the keyboard if the target road name appeared on the left side, otherwise they had to press the “RIGHT” button if it appeared on the right side. In order to assure the accuracy of the test, the designed targets were allocated on the left or right side of traffic sign. In this test, stimulus presentation time was set as 3500 ms. If participants had no reaction in 3500 ms, the displayed traffic sign disappeared and another one was presented.

### 2.5. Experimental Procedure

Before the experiments, all subjects gave their informed consent for inclusion before they participated in the study. The study was conducted in accordance with the Declaration of Helsinki, and the protocol was approved by the Academic Committee of Intelligent Transportation System Research Center, Wuhan University of Technology. The formal experimental procedure was as follows:

(1) Before the test, subjects were asked to complete a questionnaire providing their basic information: age, gender, driving experience, whether myopic or not, whether the subject had underwent a brain operation, whether the subject had a cold, and whether the subject drank coffee or other stimulating drinks and drugs that would affect brain function before the test. All subjects that did not meet the conditions were removed. At the same time, subjects were required to provide their signatures for the reward.

(2) The staff explained the test procedures and ensured that all subjects understood the test requirements and contents before practice. Silence must be maintained in the control room and all communication tools should be turned off.

(3) Before the experiment, subjects were instructed to turn off all mobile phones and other communication tools. They were reminded to choose a comfortable posture, to try to relax during the experiments, and try their best to refrain from moving their body and head.

(4) The experiment instructions shown on the screen was: “This is a visual workload test. At the beginning of the test screen, a ‘+’ character will appear. Then, there will be a road name, which will disappear after three seconds. After that, a ‘+’ will appear and a number of signs will be presented in sequence. Find the previously shown target road name in the traffic signs and press the right key based on your judgment of the road name’s location. If the target path name appears on the left side of the sign, press the left key; if the target path name appears on the right side of the sign, press the right key.”

(5) At the end of the experiment, all participants were asked to complete a questionnaire of subjective workload with approximately 22 typical signs, in order to test the amount of information that correlated between theoretical calculation and subjective evaluation.

### 2.6. Results

#### 2.6.1. Relationship between Objective Information Amount and Workload Subjective Rating

**Figure 3:**
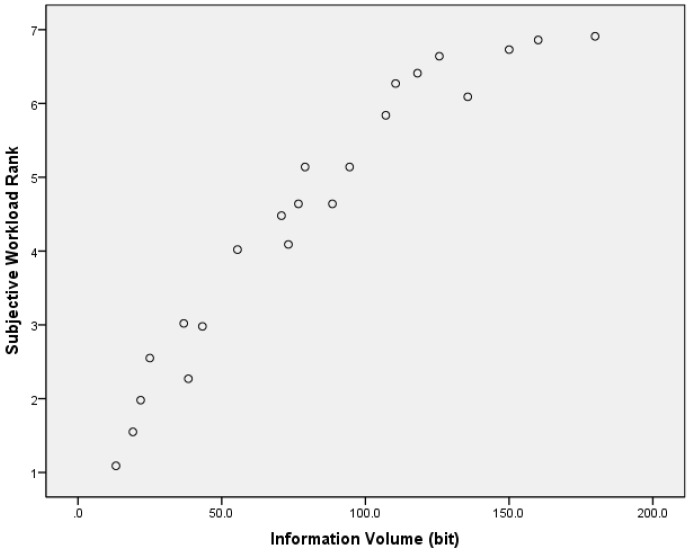
The correlation between objective information and subjective score.

In order to verify the rationality of information quantization, the participant’s workload subjective score of the traffic sign within different information grades were analyzed. The subject workload rank on a scale from 1 to 7, which was transferred from NASA-TLX, where number 1 represents the lowest information workload, while number 7 represents the highest information workload. The result is considered as an information-subjective evaluation. The average value of the scores of all drivers is the final result of the subjective information workload, while the amount of information obtained using Equation (3) is called the objective information amount. The amount of subjective information workload and objective information of the 22 traffic signs are shown in Figure 3.

The objective information and subjective workload rank correlation analysis show that the correlation coefficient is *r* = 0.958 (*p* < 0.01). This indicates that the amount of the information calculation method and the drivers’ subjective information workload matches well. Therefore, we can use the information volume of traffic signs to calculate and evaluate the workload caused by traffic signs.

#### 2.6.2. Driver’s Cognition Time under Different Information Levels

Regarding traffic sign visibility and cognitive workload, studies have focused on reaction time. Reaction time is an important index for cognitive workload. Reaction time also plays an important role in practical applications, because traffic accidents are partly caused by the insufficient reaction time of the driver. A longer reaction time means that a driver has to use more mental capacity for traffic sign cognition, which produces a greater workload. Since signs within the same information level appeared several times during the test, the average reaction time of a certain information level was regarded as a subject’s reaction time.

**Figure 4:**
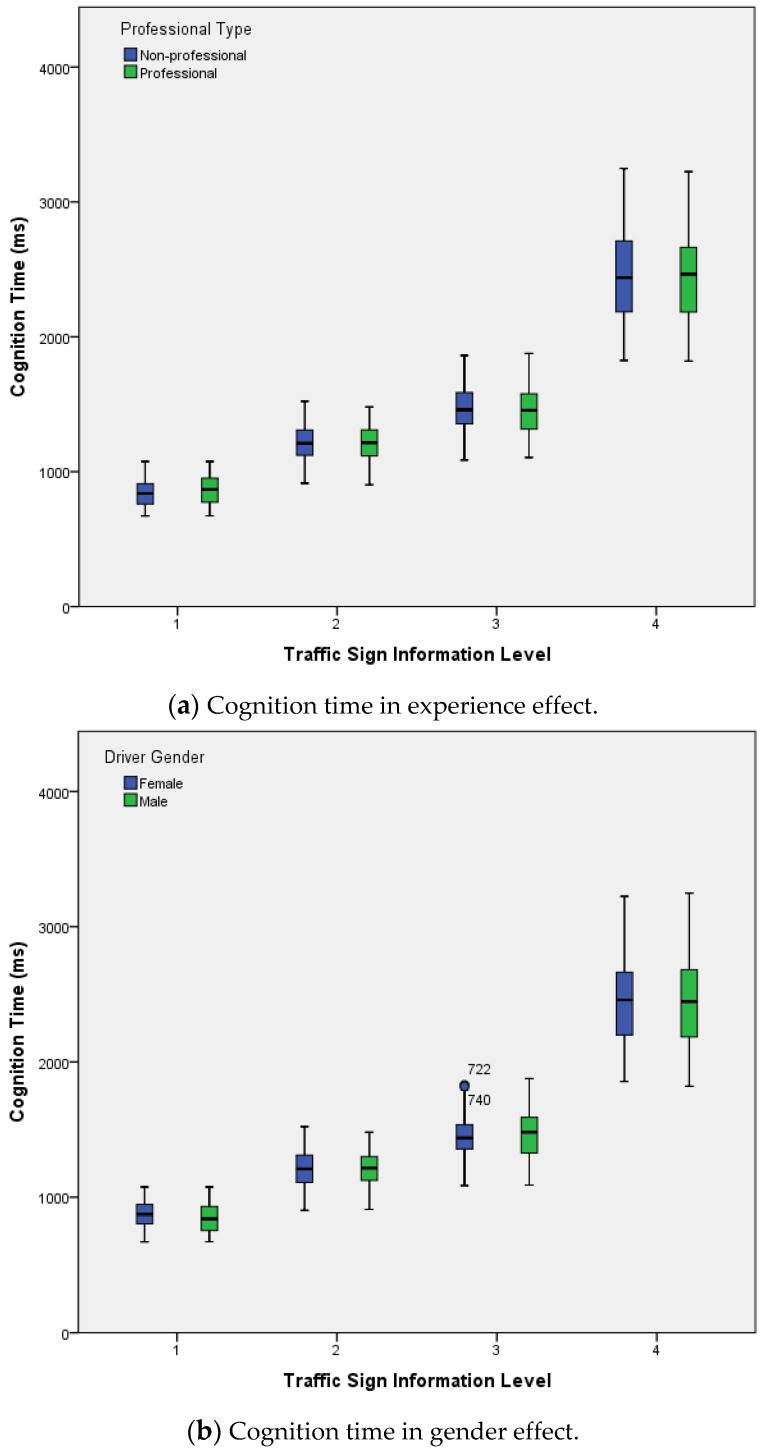
The reaction time of subjects to signs in different levels of information.

Descriptive statistical results for the cognition time of subjects to traffic signs in different information level. MANOVA results in different situation are showed in Table 2 and Table 3. Merely information significantly influenced cognition time (*F* = 2130.3, *p* < 0.001), while there was no experience (*F* = 0.7, *p* = 0.393), gender (*F* = 0.2, *p* = 0.644), information interaction with experience (*F* = 0.2, *p* = 0.917), experience interaction with gender (*F* = 2.1, *p* = 0.147), information interaction with gender (*F* = 0.4, *p* = 0.779), nor information, experience interaction with gender (*F* = 1.0, *p* = 0.384) effect on cognition time. The average cognition time was lowest at Level 1 information volume (Mean = 856.4, SD = 105.2), and the highest average cognition time was at Level 4 information volume (Mean = 2444.4, SD = 336.4), as shown in Figure 4.

#### 2.6.3. Relationship between Traffic Sign Information and Cognition Workload Rank

Previous studies have shown that different volumes of traffic sign information will cause different visual cognitive difficulties when a driver recognizes a traffic sign [34]. Visual cognitive difficulty can be regarded as a kind of workload [35]. In this study, the relationship between workload subjective evaluation grades and traffic signs with different amounts of information was analyzed. When conducting this experiment, the subjects were required to complete the visual recognition workload (difficulty) questionnaire on the signs. Workload level was graded based on subjective evaluation results. Then, the relationship between traffic sign information and cognition difficulty were discussed. Table 4 and Table 5 shows the descriptive statistical results for subjective workload rank and MANOVA results for the difference between factors. Information Level (*F* = 1137.3, *p* < 0.001) and information×experience (*F* = 5.4, *p* < 0.05) significantly influenced the Subjective Workload Rank of traffic signs with different information volumes, while there was no experience (*F* = 2.8, *p* = 0.097) , gender (*F* = 0.8, *p* = 0.377), experience interaction with gender (*F* = 0.2, *p* = 0.665), information interaction with gender (*F* = 0.5, *p* = 0.674), nor information, experience interaction with gender (*F* = 1.0, *p* = 0.384) effect on subjective workload ranks.

**Figure 5:**
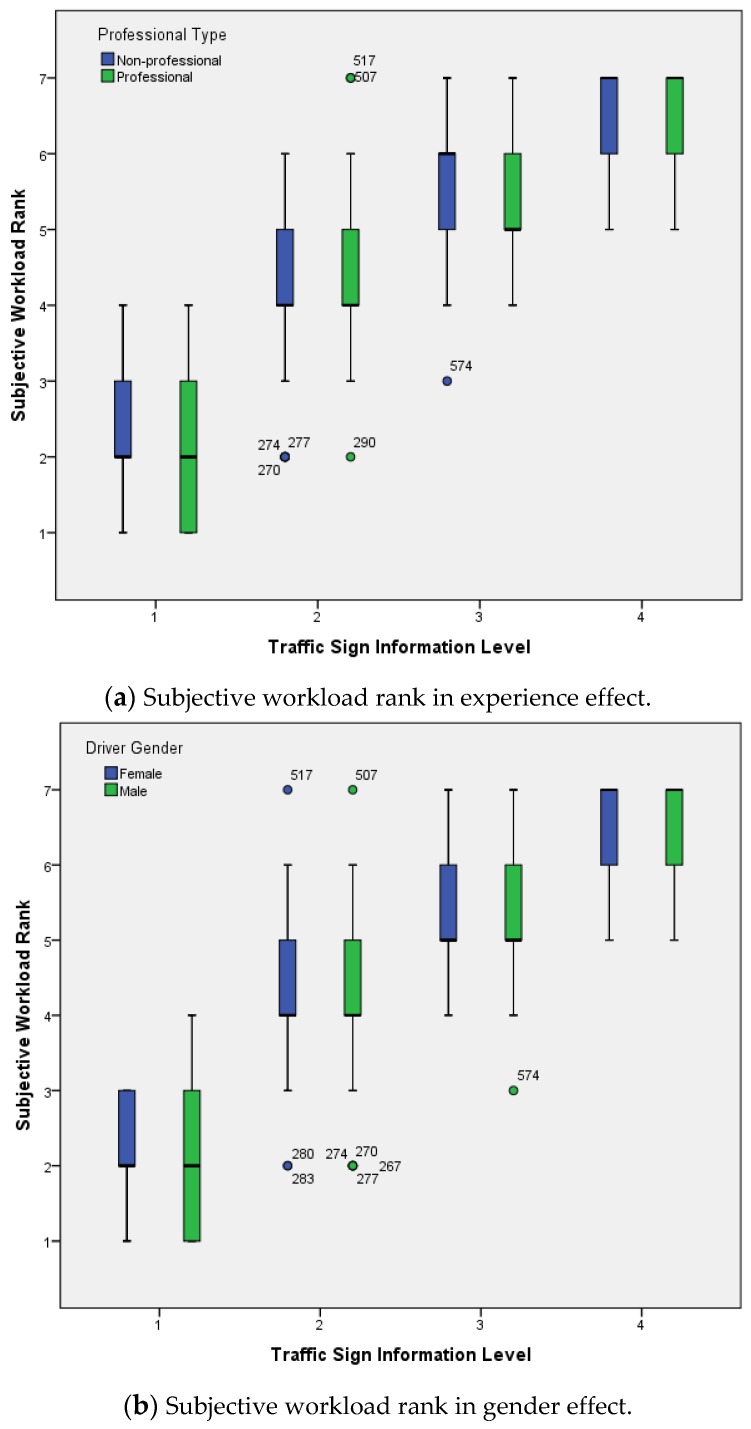
Subjective cognitive workload rank under different information levels.

The average subjective workload rank was lowest at Level 1 information volume (Mean = 2.07, SD = 0.787), and the highest average subjective workload rank was at Level 4 information volume (Mean = 6.56, SD = 0.574), as shown in Figure 5.

## 3. Study 2: Driving Performance under Workload

A driver’s driving performance such as speed control and lane keeping ability are reflections of how well a driver adapts to his or her workload. Therefore, the quantification of driving workload and the research on a driver’s behavior can be achieved by analyzing driving performances. A driving simulator can create a higher workload scene for use in implementing tests. In addition, the Scene Editor can generate all kinds of workload scenes, such as traffic signs with different amounts of information and curves with different radii. Meanwhile, it is easy to measure a driver’s operating performance.

In this part, the driving behavior and performance of the traffic sign information load in highway ramp sections were studied from the view of a driver’s mental load. Experiments were designed and carried out for issues such as its impact on traffic safety caused by information workloads of highway ramp sections. We collected the manipulation data and response data for guiding signs of the on simulation platform, and comparatively analyzed the operating performance and time of cognizing signs in different driving workloads. For speed controlling, lane keeping was analyzed at different cognitive workload levels. The study and its conclusions are instructive for guiding the quantitative research of road traffic sign information and designing driving workload experiments.

### 3.1. Subjects

The experiment was a 4 (information level) × 2 (driving experience) × 2 (gender) repeated measures mixed design. Forty-four participants were recruited and drivers participated both in Study 1 and Study 2.

### 3.2. Apparatus

Driving simulators have been widely used in this field, as in traffic safety. The main idea was to obtain an estimate of the driving performance, the corresponding results on real roads through a driver’s reaction, and the corresponding data under simulated driving conditions. This is safe, and repeatable and the conditions can be easily controlled. In this study, the tests reflected a driver’s driving safety levels in different driving workload conditions, which was mainly based on the data of a driver’s simulated driving operations.

The research-based comprehensive driving simulator for traffic safety was established by the Intelligent Transportation Systems Research Center of Wuhan University of Technology. It is mainly directed to road traffic studies, which is a set of studies of a driver’s behavior characteristics, driver’s training and testing, research for traffic control and simulative research for automatic driving. In addition, the simulator can be used for traffic studies in the lab, which can offer a reliable experimental platform, abundant traffic conditions, and different scenes.

**Figure 6:**
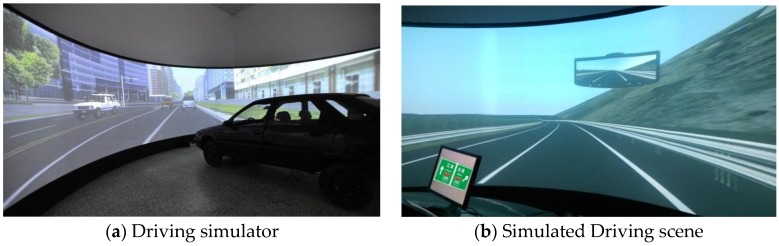
Driving simulator and a scene.

Multi-channel driving simulation platforms have integrated automotive control, computer simulation, and image fusion technology by using advanced techniques that can create a strong sense of reality and an immersing sense of operation, and simulate real traffic scenes. Its basic structure consists of a projection system, sound system and vehicle unit. The projection system consists of a projector, image fusion machine, graphic workstation, sound equipment and circular-screen system. The vehicle unit consists of sensors for collecting driving operation data, cockpit, navigation equipment, and signal transmission devices. The actual effect of the driving simulator is shown in Figure 6. The signs are shown using a 23’ LED screen situated right front of the simulator, and the E-prime software was used to control the display of the traffic signs.

### 3.3. Driving Simulation Experiment Design

Traffic signs were used to specify different levels of driving workload. The driving simulator experiment was conducted as a 4 (information level) × 2 (driving experience) × 2 (gender) repeated measures mixed design. The experience and gender were between-subjects factors and traffic sign information levels was the within subjects factor. The dependent variables were driving speed and lateral offset of the vehicle.

Firstly, it is necessary to explain the testing procedure and tasks to the subjects. It is helpful for the subjects to familiarize themselves to the testing environment before the test to ensure the quality of the results. After ensuring all the devices are in working condition, the test was started, and the data of the different devices would be synchronized at the same time. The main experimental data are the drivers’ performance information. These were collected by the driving simulator system.

In the simulated driving test, each subject should respectively finish the secondary task of recognizing traffic signs while executing the driving primary task. Different signs contain different amounts of information. Based on the information amount calculation method mentioned in Fu’s previous research [9], the amount of information in different signs was calculated. The amount of information in different signs was graded using statistical analysis. Grading was divided into four intervals: (0, 40), (40, 80), (80, 120) and (120, +∞).

The testing scenes mainly involved a highway and its ramp sections. The designed speed was 100 km/h, two-way with 6-lanes, cross slope was 3%, and the maximum longitudinal slope was 2%. The designed testing scene was circular with total length of 10 km. This can support continuous driving for the testing vehicle. The driving scene consists of prepared roads and testing roads. Prepared roads are 2-km long, which is the vehicle’s start of every test. The main purpose was used for the vehicle’s acceleration; namely, accelerating from standstill to the desired speed. The road’s radius of the horizontal curve was 1200 m, the easement curve A = 80, and the radius of the vertical curve was 1800 m. All alignment combinations comply with the design demand of “horizontal include vertical” in road engineering.

The traffic signs are set in front of the off ramps at 200 m. In the tests, the prepared notification signs are not set. One reason is that the test needs to measure the subjects’ processing and recognition of the new sign information.

The signs selected for testing are typical types of each driving workload level, which are set in the highway ramp sections. In the test, the subject participants were told the name of a certain destination road, and the drivers drove in the highway scene under simulative conditions. When approaching the off ramp, the screen projected a traffic board with many destinations. The subject should either choose between driving onto the ramp or going straight by pushing the mouse buttons near the steering column. The subject should press the right mouse button if he wants to drive off the ramp, otherwise, he should press the left button. The signs that appear are controlled by the E-prime software, which can easily determining whether the reaction was right.

According to the requirements of the test, the cognitive difficulty levels of the signs are different, and the subjects should finish the corresponding primary and secondary task at the same time. In the test, the definitions of the primary task and secondary task are as follows:

(1) Primary task: maintain the driving speed and keep diving along a certain lane, and avoid changing lanes as much as possible;

(2) Secondary task: recognize the signs. The subjects should recognize the signs and react by pressing the button in response to different signs. The amounts of sign information are different.

Every driver should recognize the signs with information level 1, 2, 3 and 4, while driving at a target speed of 100 km/h; and perform judgment operations.

### 3.4. Driving Simulation Experiment Implementation

Controlling the testing conditions is very important for the quality of the data, and it is a key point that ensures the success of the test. The main factors that impact the tests are lights and sound stimulation. Lights should be kept at a constant level during the test, which can be realized by turning on the same lights. The noise that influences the subjects is controlled at the same time. In the testing site located in the driving simulation lab of Wuhan University of Technology, testing conditions can be easily controlled. The testing procedures mainly include the following steps:

(1) First, the subjects are informed about the contents and requirements of the test. They should fill in their basic information: age, gender, driving age, with myopia or not, sick or not, whether the subject drunk coffee or some simulative drinks before test, and whether the subject had a brain operation. Then, subjects that failed to meet the conditions were excluded. In addition, the subjects’ signatures were required as a condition of payment.

(2) Before the test, ensure that the data collection is working normally. Turn on the simulative driving device, choose the corresponding driving scene, and start the E-Prime software. By operating the steering and throttle of the simulator, vehicle movement such as speed and lane deviation data can be checked.

(3) The subject can perform 10-min exercises to familiarize themselves with the simulative driving environment. During the exercise, the subject was verbally given a certain place name. When approaching a ramp, the subject should choose whether to drive off the ramp or go straight based on the different destinations. If the subject wants to drive onto the ramp, the right mouse button should be pressed; otherwise, the left mouse button should be pressed.

(4) Formal test and data recording. Non-participants were instructed to wait outside the testing room, maintain silence, and turn off all their communication devices. The subjects were reminded to choose a comfortable posture. They should relax and prevent their body and head from moving. At the beginning of the test, a camera was used to record the starting time of the data collection from different devices. This would be used for analyzing the following testing data.

The formal test was divided into different blocks. These different blocks had different amounts of sign information. In each block, the experiment coordinator verbally informed the subject of the destination name. When the car drives close to the ramp, the coordinator operates the E-prime program to present the sign in the computer monitor. The subject should press the button nearby the steering as fast as possible when they see the sign information to finish the secondary task. In the test, the mouse is next to the steering wheel. Hence, it is convenient for the subject to press the button.

After finishing each block, the subjects were required to fill a subjective driving workload scale questionnaire. After finishing each test, the data should be copied and managed for the following analysis.

**Figure 7:**
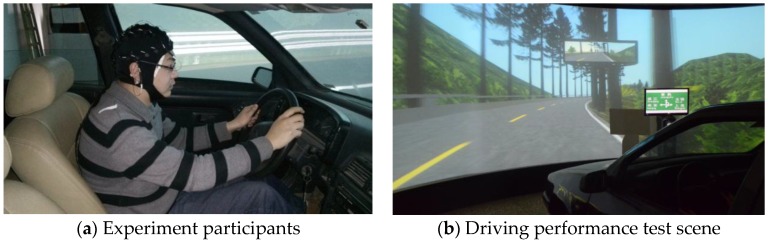
Driving workload test with physiological measurement.

It should be explained that in simulative conditions, the visibility effect of traffic signs in the scene is bad while driving. The subject should drive very close to recognize the contents of the signs, which is inconvenient for testing. In addition, it is very hard to confirm the correct time a participant would start to recognize the signs, because traffic signs keep on moving. For this situation, a LED monitor was used to present the signs in front of the simulating car. Furthermore, its height was modified to be similar to the height of signs during simulative driving. On one hand, the definition of these signs was similar to a real driving scene; on the other hand, it was easy to control the collection of the present time of the signs during the test. The test scene is shown in Figure 7.

### 3.5. Results

Driving performance data were mainly automatically obtained by the simulative vehicle. The data collected when driving included vehicle speed, lane off-set, front wheel angle, opening degree of the accelerator and clutch, and brake. This part focuses on the performance characteristics of drivers such as vehicle speed, lane keeping and reaction time.

#### 3.5.1. Vehicle Speed Maintenance Characteristic

In the test, each subject should finish two groups of tests. During the first and second tests, the time mark was selected when the driver reacted. The noise data produced by the operation such as deceleration of the ramp were removed. According to the timing nodes, the difference in speed in the test and the required speed in the whole test were counted separately. Descriptive statistical results for cruise speed in different information level and MANOVA results in different situation are shown in Table 6 and Table 7.

**Figure 8:**
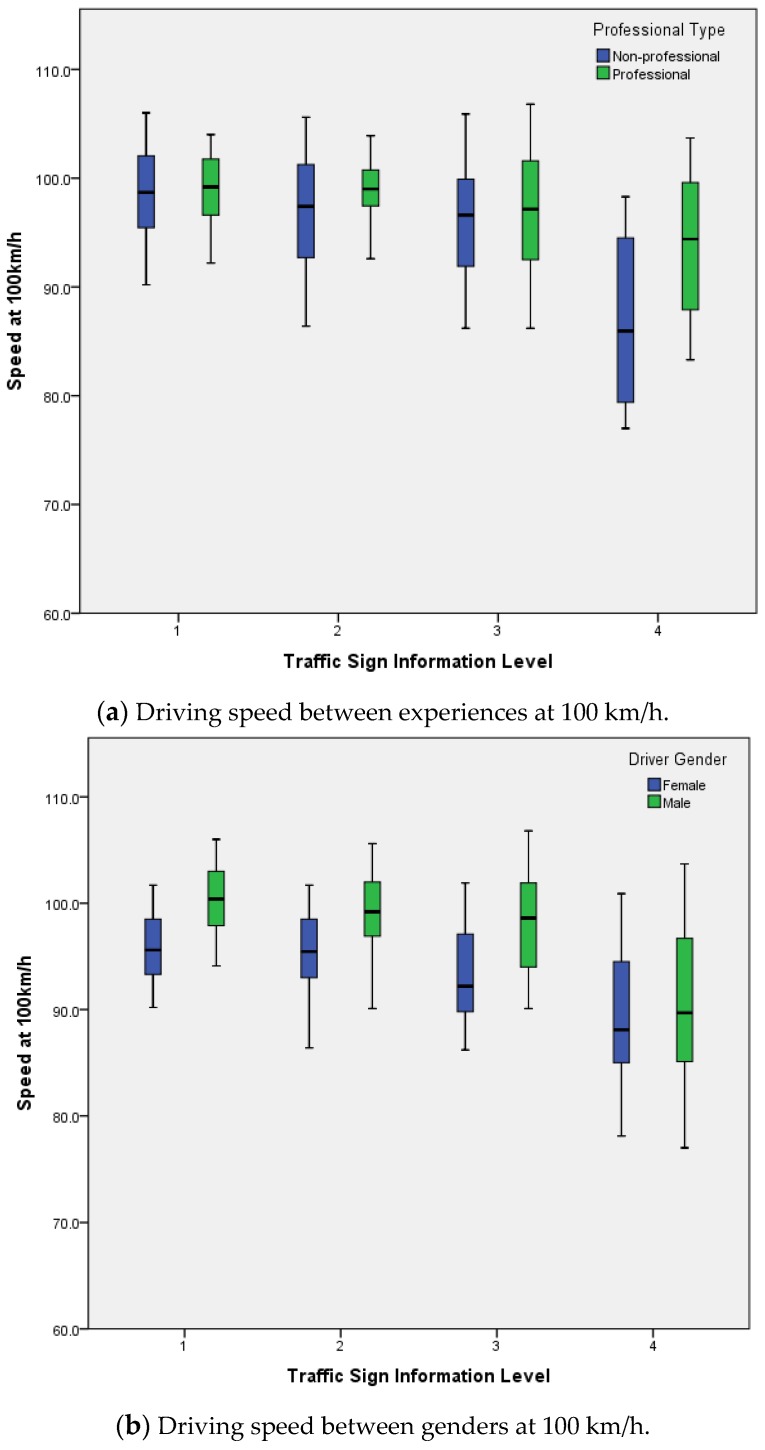
Driving speed at different sign information levels.

The results indicate that information volume significantly influenced the driving speed (*F* = 123.8, *p* < 0.001). The average driving speed was lowest at Level 4 information volume (Mean = 90.1, SD = 7.54), and the highest average driving speed was at Level 1 information volume (Mean = 98.9, SD = 3.72). There were experience (*F* = 12.7, *p* < 0.001), gender (*F* = 113.6, *p* < 0.001), information interaction with experience (*F* = 11.5, *p* < 0.001), experience interaction with gender (*F* = 27.1, *p* < 0.001), information interaction with gender (*F* = 7.3, *p* < 0.001), and information, experience interaction with gender (*F* = 5.7, *p* < 0.05) effects on driving speed. The average driving speed of an experienced driver was 97.3 (SD = 5.05), and the average driving speed of a non-professional driver was 95.0 (SD = 7.00). The average driving speed of female drivers was 93.5 (SD = 5.30), and the average driving speed of male drivers was 97.2 (SD = 6.24), as shown in Figure 8.

#### 3.5.2. Lane Keeping Performance Analysis

Since the lateral offsets are different and the data of the lateral offset will be positive and negative, only the absolute value of the data were collected before analysis. Descriptive statistical results for lane deviation in different information levels and MANOVA results in different situations are shown in Table 8 and Table 9.

Results indicate that information volume significantly influenced the lane deviation (*F* = 212.6, *p* < 0.001). The average lane deviation was lowest at level 1 information volume (Mean = 0.167, SD = 0.13), and the highest average lane deviation was at level 4 information volume (Mean = 0.575, SD = 0.322). The main effects of experience (*F* = 79.2, *p* < 0.001), gender (*F* = 214.3, *p* < 0.001) are significant, and interaction effect of information with experience (*F* = 8.1, *p* < 0.001) was significant. The interaction effect of experience interaction with gender (*F* = 0.0, *p* > 0.05), interaction effect of information with gender (*F* = 1.0, *p* > 0.05), and interaction effect of information, experience with gender (*F* = 0.1, *p* > 0.05) on lane maintenance were not significant.

**Figure 9:**
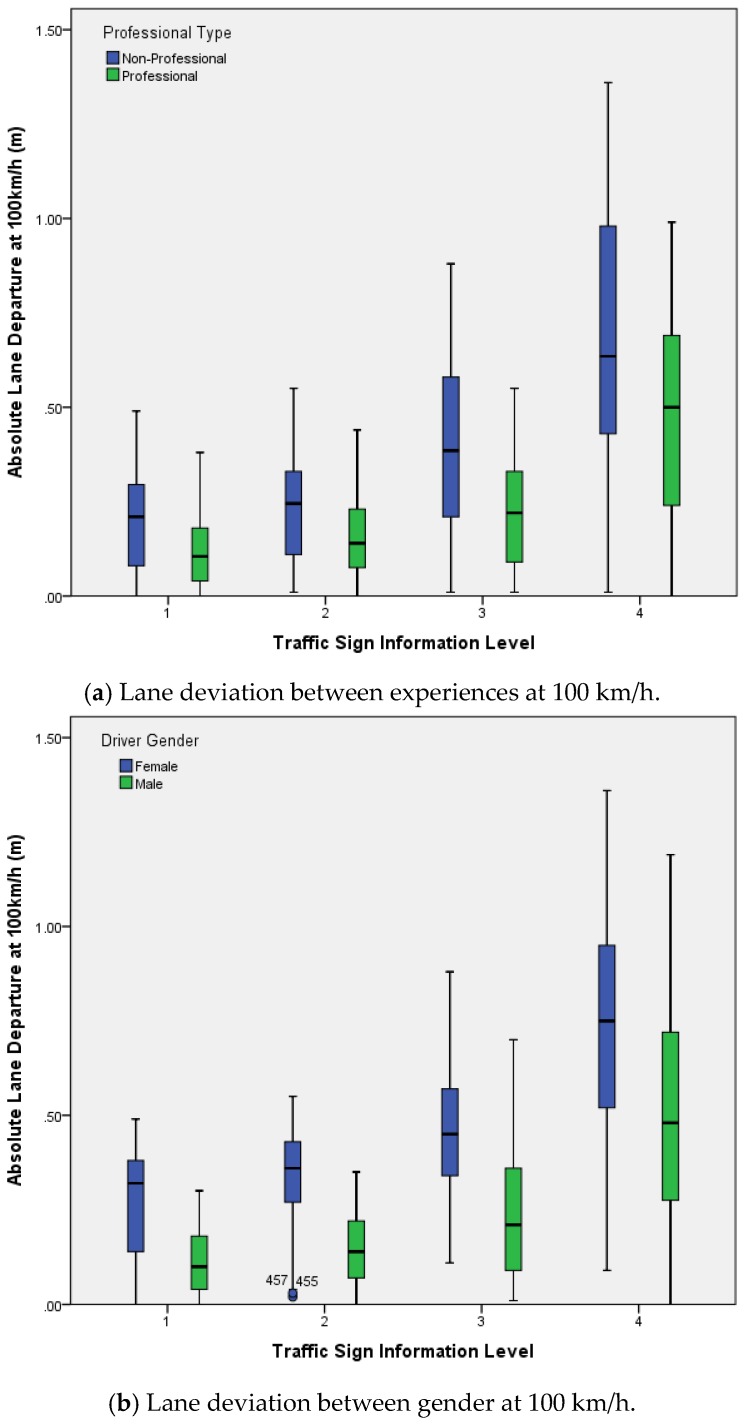
Lane deviations in different sign information levels.

The average lane deviation of experienced drivers was 0.236 (SD = 0.2075), and the average lane deviation of non-professional drivers was 0.369 (SD = 0.291). The average lane deviation of male drivers was 0.242 (SD = 0.232), and the average lane deviation of female drivers was 0.446 (SD = 0.272), as shown in Figure 9.

## 4. Discussion

### 4.1. Effect of Traffic Sign Information on Driving Workloads

Traffic sign cognition brings in a certain amount of extra driving workload, which is certain [36]. According to previous analysis, a driver’s subjective cognition difficulty varies with different amounts of information signs, and this cognition difficulty increases as the amount of information increases. Moreover, the level of sign information and its corresponding information workload subjective rating results were similar (*R*^2^ = 0.958). Therefore, the information level can be approximated as the workload level used for information workload evaluation.

Every subject’s cognition time to different levels of information signs is unequal. The average cognition time of traffic signs with levels 1–4 information are 856.4 ms, 1212.9 ms, 1462.1 ms and 2444.4 ms, respectively. Level 4 is required for most cognition times, while level 1 is required for minimum cognition time. This illustrates that signs in different amounts of information needs different amounts of efforts to cognize. This conclusion is similar to previous studies, in which cognition times to different information grade signs are quite different [34]. With regard to cognition time as an index to reflect the workload level, it is confirmed that different levels of information produce different information workloads.

Regarding the reaction time, the standard deviation of cognition time to levels 3 and 4 information is significantly greater than the value of traffic signs with levels 3 and 4 information. The main reason for this phenomenon may be due to the large amount of information contained in the signs, which would cause high driving workload. Furthermore, under high workload conditions, a driver’s reaction time would fluctuate sharply.

It was found that if only the cognitive time of traffic signs were analyzed, there would be no experience or gender effect on subjective workload rank, and no experience or gender effect on cognition time. Therefore, different information traffic signs can be used as a factor to control a driver’s information workload. The result has implications for future driving workload study. The present work provides demonstration that experiments should be designed more similar to the naturalistic driving environment. For example, it is more reasonable to using the characteristics of traffic facilities to loading workload without interference of artificial factors which just was the disadvantage of N-back tasks or numerical calculation task, etc.

Actually, traffic signs are an effective way to load workloads for a driver by increasing the amount of information, such as increasing the amount of information in traffic signs [35]. Bendak [31] used this method, and analyzed the longitudinal acceleration of a vehicle in different cognitive workloads. Significant differences between the sign sections and no sign sections were found. This illustrates that traffic sign information occupied a certain amount of mental resource, and increased the driver workload.

When the achievements of related information theory were used to quantitatively calculate road sign information, the results revealed that when the amount of road sign information exceeds a certain threshold, the driver’s visual and cognitive time would increase significantly [37]. Chinese scholars have analyzed the influence of the number of roads in a traffic sign on the driver’s searching of the target road. When the number of the roads exceeds 5 or 6, visual and cognitive time increases significantly [38]. Therefore, maintaining proper traffic sign information is significant for controlling driving workloads and improving traffic safety.

### 4.2. Effect of Workloads on Driving Performance

**Figure 10:**
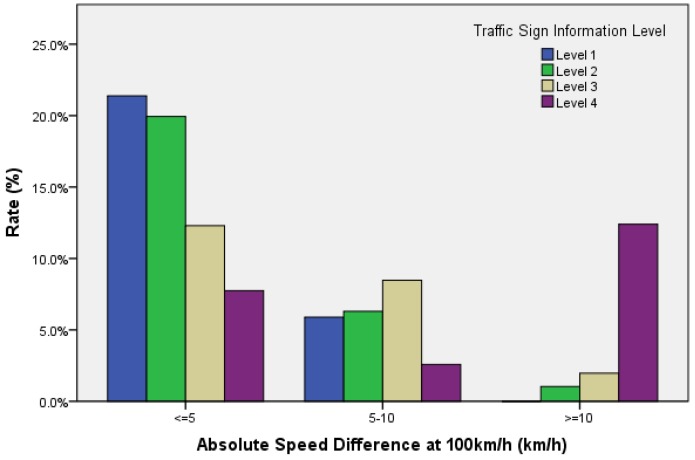
Proportion of the difference between driving speed and target speed (ΔV) in different sections.

A previous analysis in this study indicated that information volume (secondary task workload) significantly influenced driving speed. ΔV is the absolute of difference between the operating speed and the required cruise speed (100 km/h). The magnitude of ΔV reflects driver’s ability to maintaining constant speed, and it is an important driving performance indicator. The average ΔV under level 1 cognition workload was 3.2 km/h and the ΔV under level 4 was 10.3 km/h. In additional, Figure 10 indicates that a subject’s driving speed controls well while diving in a condition of low secondary task workload (Level 1 information).

The proportion of ΔV less than 5 km/h was 21.4%, and the proportion of ΔV above 10 km/h was 0.0%. While during high workloads (level 4 information), the ability of speed control is weakened. The proportion of ΔV less than 5 km/h was 7.7%, and the proportion of ΔV above 10 km/h was 12.4%. Thus, it can be explained that when driving workload increases, the difference in vehicle speed increases, and the subject’s mental resources occupied for speed keeping increases. Hence, the subject should make more efforts in speeding keeping, in order to obtain a better result. More mental workload would be occupied in order to maintain the required speed.

**Figure 11:**
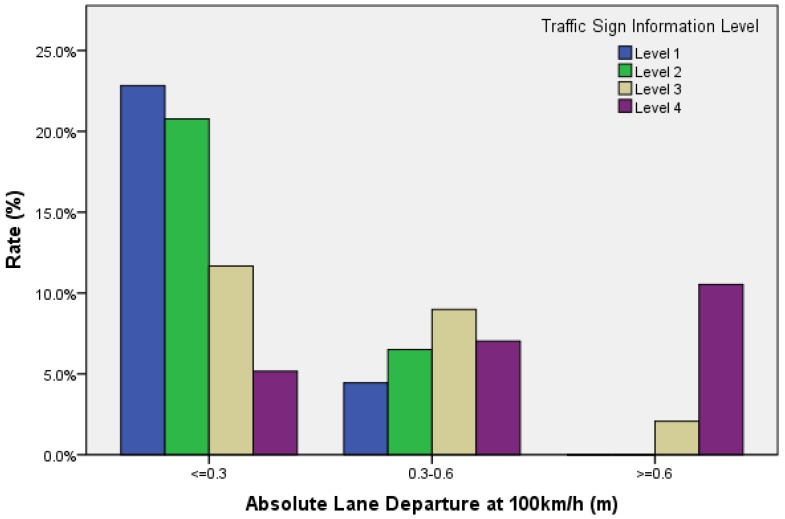
The proportion of lane deviation in different sections.

Lane maintenance mainly reflects on a subject’s ability to perform lateral control. The result in Figure 9 shows that information volume (secondary task workload) significantly influences the lane deviation characteristics. When the target speed was 100 km/h, the average lane deviation under level 1 cognition workload was 0.18 m and the lane deviation under level 4 was 0.57 m. As shown in Figure 11, when driving at level 1 cognitive workload, the proportion of lateral offset below 0.3 m was 22.8%, the proportion of lateral offset above 0.6 m was 0.0%. Furthermore, when cruising at level 4 cognitive workload, the proportion of lateral offset below 0.3 m was 5.2%, and the proportion of lateral offset above 0.6 m was 10.5%. Lane deviation reflects subjects’ ability of lane keeping. Therefore, this indicates that a drivers’ ability of lane keeping changes slightly, while the amount of information changes within certain realms; however, as lane deviation significantly increases, the ability of lane keeping significantly drops, and the amount of information increases to level 4.

**Figure 12:**
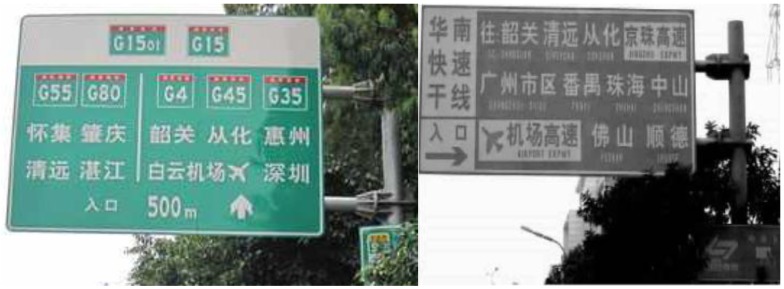
Examples of signs with information overload.

From the view of security, mandating drivers to recognize the sign completely will result in increased perception time, decreased speed and increased lane deviation, which are detrimental to driving safety. As expected, the increase in subjective driving workload and damage of drivability are led by the increase in environmental complexity. This provides a reference for traffic sign design. Abnormal traffic signs with information overload still exist in China (as show in Figure 12) due to the lack of an information capacity control standard.

As mentioned by Cao, mental workload is significantly higher in the dual-task condition compared with the single-task conditions, which related to driving safety issue [39]. In a high workload driving environment, when drivers make efforts to perform a task, for example protect lateral control against the risk of distraction, they will neglect other elements of driving performance. By research of workload mechanism, educational efforts can be applied to help drivers understand the risks of distraction and the inadequacies of compensatory driving strategies [40].

The complex influence mechanism of the traffic environment and driving workload affect driving performance. The following conclusion can be put forth: (1) generally, subjective driving workload increases due to complicated driving conditions, which would certainly lead to driving performance impairment; (2) if the complicated environment is acceptable to the skilled driver, compensation strategy is usually used to reduce the driver’s speed, which may help them achieve better driving performance; (3) a driver’s subjective mental load level increases with the increase in environmental complexity, but there are less driving performance impairment for skilled drivers; (4) even under the same complicated driving conditions, the degree of driving performance impairment exhibited by drivers were not the same in different tasks and stages (such as different stages for merging and demerging).

### 4.3. Experience and Gender Effect on Driving Performance

Driving workload is higher for new drivers than for skilled drivers. New drivers need to pay more attention while driving [23]. When the target speed was 100 km/h the results indicate that there are experience and gender effects on driving speed and lane deviation. The average driving ΔV of experienced drivers was lower than that of non-professional drivers. The average driving ΔV of male drivers was lower than that of female drivers. The average lane deviation of experienced drivers and male drivers was lower than that of non-professional drivers and female drivers. This result is similar to other data from other reports [20,41].

Research has shown that the distribution of driving task information processing patterns on the three levels can be mediated by driving experience. New drivers have a lower level of mechanical operation and executive ability [27], as their mechanical operation ability is obtained with the accumulation of actual operand. Therefore, a new driver’s high workload level is led by the driving task. Therefore, workloads obtained from operation and vehicle skills need lower driving workloads, and the driving load is quite low for a skilled driver. In addition, accident risks are 2–4 times for new drivers, compared with skilled drivers; which can be explained by the subjective safety model [25]. This model shows that the driving adaptive ability is related with environmental and driver characteristics. It particularly depends on the ability of perceived environmental complexity, mission requirements and adaptation to the workload. Moreover, a novice would usually mistakenly estimate the environment, and the compensation strategy is also relatively slow [26].

## 5. Conclusions

In this study, drivers’ cognitive behavior, especially driving performance characteristics, under different Chinese traffic sign environments were studied. Cognitive workload was applied by reading traffic signs with different information. These were divided into four levels, and were presented by the E-prime software. The drivers were required to maintain the target speed (100 km/h) while driving along designed highway off-ramps. While driving, the driving performances were recorded; and questionnaires on objective workload were collected after driving. These experiments were designed and conducted for issues such as the impacts on traffic safety caused by information workload in highway off-ramp sections. The manipulation data and response data for the traffic signs of the participants were collected on the simulation platform, and driving performance indicators under different driving workloads were comparatively analyzed.

According to the study of the data obtained from the simulated driving test, some results can be presented: (1) cognition difficulty increases as the amount of information increases. Moreover, traffic sign information level and subjective workload rank were similar. There was no experience or gender effect on subjective workload rank and cognition time. Therefore, different information traffic signs can be used as a factor to control a driver’s information workload; (2) Information volume (secondary task workload) significantly influenced driving speed and lane deviation, which indicates that driving workload has an effect on driving performance; and exceeding workloads would cause driving performance impairment; (3) There were experience and gender effects in driving speed and lane deviation. Experienced drivers and male drivers have lower driving speed and lane deviation than non-professional drivers and female drivers.

Actually, in complex traffic environments, human cognition and behavior while driving are closely related to both psychological and physiological characteristics. This also affected the dynamic environment, which involves cognitive science, behavioral scientific knowledge, human factors, and so on. Determining a reasonable level of driving load is a problem that needs to be solved at present. With the development of sophisticated modeling techniques and cognitive theory of cognitive psychology basis, the study of psychological mechanisms on the driving process using modeling has become the new trend in driving behavior research. Such studies would help integrate previously fragmented studies, and propose a theory of cognitive decision making and driving behavior. At present, perfect continuous development is mainly in cognitive architecture ACT-R [42,43]. If cognitive psychology modeling could be used into the field of traffic safety for high workload driving conditions and driver behavior modeling studies, it would be possible to understand the relationship between driving behavior and driving environments, providing a theoretical basis for ensuring driving safety.

## Figures and Tables

**Table 1:** Weight of six kinds of elements on road traffic signs.

Elements	Chinese	Symbol	Arrow	Number	English	Color	Total
Weight	0.25	0.28	0.26	0.09	0.07	0.05	1.00

**Table 2:** Descriptive statistical results for reaction time.

Source	Factors	Sample Size	Parameter	Cognition Time (ms)
Information Level	Level 1	N = 264	Mean	856.4
			SD	105.2
	Level 2	N = 264	Mean	1212.9
			SD	133.5
	Level 3	N = 220	Mean	1462.1
			SD	177.1
	Level 4	N = 220	Mean	2444.4
			SD	336.4
Experience	Non-professional	N = 22	Mean	1450.4
			SD	616.6
	Professional	N = 22	Mean	1454.0
			SD	612.2
Gender	Female	N = 13	Mean	1454.9
			SD	36.3
	Male	N = 31	Mean	1451.1
			SD	23.5
	Total	N = 44	Mean	1452.2
			SD	614.1

**Table 3:** Analysis of variance results for reaction time.

Source	d.f.	*F*-Ratio	Partial Eta Squared
Information Level	3	2130.3 **	0.807
Experience	1	0.7	0.001
Gender	1	0.2	0.000
Information × Experience	3	0.2	0.001
Experience × Gender	1	2.1	0.002
Information × Gender	3	0.4	0.001
Information × Experience × Gender	3	1.0	0.003

** *p* < 0.01, Note: Corrected Model R Squared = 0.893 (Adjusted R Squared = 0.891).

**Table 4:** Descriptive statistical results for subjective workload rank.

Source	Factors	Sample Size	Parameter	Subjective Workload Rank
Information Level	Level 1	N = 264	Mean	2.07
			SD	0.787
	Level 2	N = 264	Mean	4.22
			SD	0.897
	Level 3	N = 220	Mean	5.44
			SD	0.897
	Level 4	N = 220	Mean	6.56
			SD	0.574
Experience	Non-professional	N = 22	Mean	4.611
			SD	0.037
	Professional	N = 22	Mean	4.535
			SD	0.037
Gender	Female	N = 13	Mean	4.49
			SD	1.889
	Male	N = 31	Mean	4.42
			SD	1.842
	Total	N = 44	Mean	4.44
			SD	1.855

**Table 5:** Analysis of variance results for subjective workload rank.

Source	d.f.	*F*-Ratio	Partial Eta Squared
Information Level	3	1137.3 **	0.728
Experience	1	2.8	0.003
Gender	1	0.8	0.007
Information × Experience	3	5.4 *	0.017
Experience × Gender	1	0.2	0.000
Information × Gender	3	0.5	0.002
Information × Experience × Gender	3	1.0	0.003

** *p* < 0.01, * *p* < 0.05. Note: Corrected Model R Squared = 0.817 (Adjusted R Squared = 0.814).

**Table 6:** Descriptive statistical results for driving speed under workload.

Source	Factors	Sample Size	Parameter	Speed
Information Level	Level 1	N = 264	Mean	98.9
			SD	3.72
	Level 2	N = 264	Mean	97.8
			SD	4.16
	Level 3	N = 220	Mean	96.6
			SD	5.06
	Level 4	N = 220	Mean	90.1
			SD	7.54
	Total	N = 968	Mean	98.9
			SD	3.72
Experience	Non-professional	N = 22	Mean	95.0
			SD	7.00
	Professional	N = 22	Mean	97.3
			SD	5.05
Gender	Female	N = 13	Mean	93.5
			SD	5.30
	Male	N = 31	Mean	97.2
			SD	6.24
	Total	N = 44	Mean	96.1
			SD	6.20

**Table 7:** Analysis of variance results for driving speed.

Source	d.f.	*F*-Ratio	Partial Eta Squared
Information Level	3	123.8 **	0.281
Experience	1	12.7 **	0.013
Gender	1	113.6 **	0.107
Information × Experience	3	11.5 **	0.035
Experience × Gender	1	27.1 **	0.028
Information × Gender	3	7.3 **	0.023
Information × Experience × Gender	3	5.7 *	0.018

** *p* < 0.01, * *p* < 0.05. Note: Corrected Model R Squared = 0.459 (Adjusted R Squared = 0.451).

**Table 8:** Descriptive statistical results for lane deviation under workloads.

Source	Factors	Sample Size	Parameter	Lane Deviation
Information Level	Level 1	N = 264	Mean	0.167
			SD	0.130
	Level 2	N = 264	Mean	0.201
			SD	0.138
	Level 3	N = 220	Mean	0.314
			SD	0.213
	Level 4	N = 220	Mean	0.575
			SD	0.322
Experience	Non-prof.	N = 22	Mean	0.369
			SD	0.291
	Professional	N = 22	Mean	0.236
			SD	0.207
Gender	Female	N = 13	Mean	0.446
			SD	0.272
	Male	N = 31	Mean	0.242
			SD	0.232
	Total	N = 44	Mean	0.302
			SD	0.261

**Table 9:** Analysis of variance results for lane deviation.

Source	d.f.	*F*-Ratio	Partial Eta Squared
Information Level	3	212.6 **	0.401
Experience	1	79.2 **	0.077
Gender	1	214.3 **	0.184
Information×Experience	3	8.1 **	0.025
Experience×Gender	1	0.0	0.000
Information×Gender	3	1.0	0.003
Information×Experience×Gender	3	0.1	0.000

** *p* < 0.01, Note: Corrected Model R Squared = 0.547 (Adjusted R Squared = 0.540).
